# Comparing fluorescence-based cell-free assays for the assessment of antioxidative capacity of high-density lipoproteins

**DOI:** 10.1186/s12944-016-0336-y

**Published:** 2016-09-22

**Authors:** Fumiyoshi Tsunoda, Stefania Lamon-Fava, Katalin V. Horvath, Ernst J. Schaefer, Bela F. Asztalos

**Affiliations:** From the Cardiovascular Nutrition Laboratory, Jean Mayer USDA Human Nutrition Research Center on Aging, Tufts University, 711 Washington Street, Boston, MA 02111 USA

**Keywords:** High density lipoprotein, Oxidation, Coronary heart disease, Fluorescence assay

## Abstract

**Background:**

Population studies have shown an inverse association between high-density lipoprotein (HDL) cholesterol levels and risk of coronary heart disease (CHD). HDL has different functions, including the ability to protect biological molecules from oxidation. Our aim was to evaluate the performance of two fluorescence-based assays in assessing the antioxidative capacity of HDL.

**Methods:**

We compared the antioxidative capacity of HDL with the phospholipid 2’,7’-dichlorodihydrofluorescein (DCF) assay and the dihydrorhodamine 123 (DHR) assay in controls and in subjects at increased risk of CHD, including subjects with established CHD, and subjects with elevated plasma triglycerides (TG), serum amyloid A (SAA), or myeloperoxidase (MPO) levels.

**Results:**

The antioxidative capacity of HDL, as measured by the DCF assay, was significantly lower in both CHD and high-TG patients than in controls (*p* < 0.001 and *p* = 0.004, respectively). Interestingly, the mean antioxidative capacity of HDL in high-SAA subjects was significantly higher (*p* < 0.03), while in high-MPO subjects was similar to controls. When the DHR assay was used we did not find differences in HDL’s antioxidative capacity between CHD patients and controls but we found higher antioxidative capacity in high-SAA subjects compared to controls.

**Conclusions:**

Only the DCF assay could detect significant differences in the antioxidative capacity of HDL between controls and CHD subjects. Practical use of both assays for the assessment of antioxidative capacity of HDL is limited by the large overlap in values among groups. The antioxidative activity of HDL in patients who have elevated SAA levels needs to be reassessed.

## Background

The epidemiological evidence of an inverse association between plasma high-density lipoprotein cholesterol (HDL-C) levels and risk of cardiovascular disease is well established [1, 2]. HDL can protect against the development and progression of atherosclerosis by promoting reverse cholesterol transport (RCT), maintaining endothelial function, and reducing inflammation and oxidation [3]. In subjects who had undergone assessment of cardiovascular disease by coronary angiography or carotid intima-media thickness, the cholesterol efflux capacity of HDL was shown to be inversely associated with the extent of cardiovascular disease, independent of plasma HDL-C and apolipoprotein A-I (apoA-I) levels [4]. However, it has also been shown that inflammatory conditions or oxidative stress may impair the protective functions of HDL [5, 6]. In subjects receiving a low-dose endotoxin injection, a reduction in the functional capacities of HDL that was not consistent with the changes in HDL-C or apoA-I levels has been observed [7]. Therefore, it is now suggested that measurement of HDL functional capacity may be a better predictor of cardiovascular disease risk than HDL-C levels. Navab et al. [8] and Kelesidis et al. [9] have developed cell-free assays to measure the antioxidative activity of HDL, one of the functional capacities of HDL, using fluorescence probes. A number of studies using these assays have shown an impaired antioxidative activity of HDL in patients with both acute and chronic inflammatory disorders such as acute coronary syndrome (ACS), systemic lupus erythematosus, and diabetes [5, 6, 10]. In these studies, although the assays could detect differences between control subjects and patients, there was considerable overlap between the groups and large standard deviation of the mean values.

To validate the performance of the fluorescence probe-based antioxidative assays, we sought to determine whether these assays could detect differences between apparently healthy subjects and patients whose conditions are thought to impair the antioxidative activity of HDL.

## Methods

### Subjects and samples

Venous blood samples were collected from 160 apparently healthy subjects (controls), 98 patients with cardiovascular disease, 37 subjects with elevated triglyceride (TG) levels (>250 mg/dL), 40 subjects with elevated serum amyloid A (SAA) levels (>300 mg/L), and 30 subjects with elevated serum myeloperoxidase (MPO) levels (>500 pmol/L). Mean age of control and CHD subjects was 58 ± 16 and 61 ± 7 years, respectively. Control subjects had normal plasma total cholesterol (TC), TG and low-density lipoprotein cholesterol (LDL-C) levels (less than 200, 150, and 160 mg/dL, respectively). Subjects with cardiovascular disease had established coronary heart disease (CHD), defined as history of previous MI, stable angina, a catheterization report of ≥50 % stenosis of at least one coronary artery, abnormal exercise tolerance test or nuclear imaging procedure, or angioplasty. Hypertension was present in 4.5 % of controls and in 48 % of CHD subjects and type 2 diabetes in 3.4 % of controls and 19 % of CHD subjects. Also, hypertension was present in 13 % of high TG, 10 % of high SAA and 11 % of high MPO subjects, while diabetes was present in 9 % of high TG, 10 % of high SAA and 6 % of high MPO subjects.

Plasma and serum samples were obtained by centrifugation and immediately stored at −80 °C until measurement. Only plasma or serum were available in some study subjects, therefore, appropriate plasma and serum controls were used. The HDL antioxidative activity was measured in different biological samples: HDL fractions obtained by ultracentrifugation (UCHDL), apoB-depleted (apoBd) plasma, or apoBd serum. ApoBd plasma (or serum) samples were obtained by adding 0.4 parts of a polyethylene glycol (PEG) 6000 solution (Qiagen, Valencia, CA) (20 % PEG in 200 mM glycine buffer, pH 7.4) to 1 part plasma (or serum) followed by centrifugation at 10,000 *g* for 30 min at 4 °C. ApoBd samples were assayed within one hour. UCHDL samples were separated by density-gradient ultracentrifugation (density = 1.063–1.210 g/mL) followed by dialysis at 4 °C. UCHDL samples were assayed within three hours after dialysis.

### Laboratory measurements

Total cholesterol, low-density lipoprotein cholesterol (LDL-C), HDL-C, TG, and apoA-I levels were measured using automated standardized assays from Roche (Indianapolis, IN). Small-dense LDL-C (sdLDL-C) levels were measured using kits provided by Denka-Seiken (Tokyo, Japan) [11]. SAA and MPO levels were measured using kits from Siemens on a Dimension and a BNII clinical chemistry system, respectively. The apoA-I-containing HDL subpopulation profile was determined by non-denaturing two-dimensional gel electrophoresis as previously described [12]. High sensitivity C reactive protein (hs-CRP), glucose and insulin levels were measured in a central laboratory using established assays.

### Measurement of the antioxidative activity of HDL

The antioxidative activity of HDL was measured by two different fluorescence-based cell-free assays.

The antioxidant capacity of HDL was measured in 269 subjects by Vascular Strategies (Plymouth Meeting, PA) using the organic phospholipid 2’,7’-dichlorodihydrofluorescein (DCF) diacetate probe, as previously described by Navab et al. [8]. This assay is based on the inhibition of oxidation of the fluorescence probe by HDL. Briefly, apoBd plasma or serum samples were incubated with oxidized LDL and DCF diacetate. In the presence of oxidized LDL, DCF diacetate was converted to DCF, its fluorescence form. The fluorescence excitation/emission was measured at a 485/528 nm for one hour. To calculate the antioxidant value of HDL of each sample, the fluorescence intensity was divided by a reference value. This ratio is commonly referred to as the HDL inflammatory index (HII) [8]. A value > 1.0 indicates dysfunctional (pro-oxidant) HDL, while values < 1.0 indicate normal (antioxidant) HDL. The second assay was used in our laboratory to determine the antioxidative activity of HDL using the dihydrorhodamine 123 (DHR) as the fluorescence probe [9]. DHR (Life Technologies, Gaithersburg, MD) was dissolved in dimethyl sulfoxide (DMSO) to a 50 mM DHR stock concentration. Iron-free buffered saline was prepared as follows: 60 nM of deferoxamine mesylate (Sigma-Aldrich, Saint Louis, MO) was added to HBS buffer (HEPES 20 mM, NaCl 150 mM, pH 7.4) followed by treatment with 1.0 g/dL of Chelex® 100 resin (Bio-Rad, Hercules, CA), as previously described [13]. The concentrated DHR stock reagent was diluted 1:1000 in the iron-free HBS buffer to the 50 μM DHR working solution just before use. Five μg of HDL-C of either UCHDL or apoBd samples was added to each well in a 96-well flat bottom black plate (Fisher Scientific, Waltham, MA). The volume of each well was adjusted to 150 μL using the iron-free HBS buffer. Then, 25 μL of the 50 μM DHR solution was added to reach a final DHR concentration of 7 μM and the plate was immediately placed into an FLx800 Fluorescence Reader ® (BioTek, Winooski, VT). Samples were run in quadruplicate and a standard sample was added to each assay plate. The fluorescence intensity of oxidized DHR was assessed at five-minute intervals for one hour at 485/528 nm excitation/emission. The oxidative rate of DHR (ORD) was calculated as the linear regression slope of the fluorescence intensity between 10 and 60 min as calculated with Microsoft Excel software. The ORD value of each sample was determined as the mean of the quadruplicate slopes.

For both assays, the lower the number, the higher the antioxidant capacity of HDL.

### Statistical analysis

Statistical analyses were performed with the SPSS software version 22 (IBM SPSS, IBM Corporation, Somers, NY). Variables with normal distribution were expressed as mean ± SD and those with non-normal distribution were expressed as median and interquartile ranges. Differences between values among groups were tested by Mann-Whitney *U* test. Spearman correlation coefficients describe the relationships between variables. Statistical significance was accepted at *p* < 0.05.

## Results

### Antioxidant capacity of HDL as measured by the DCF assay

Analysis of antioxidative capacity of apoB-depeted (apoBd) samples, as assessed with the DCF assay, in control subjects revealed no significant differences between men and women (Table 1). Therefore, in subsequent analyses, men and women were combined in one group. However, serum samples had significantly lower antioxidative capacity than plasma samples (Table 1); therefore, in our analyses we matched for sample type.

**Table 1:** Plasma lipid levels and antioxidative capacity of HDL as measured by the DCF assay in apoBd plasma and serum samples in control subjects

	Control Subjects			
	Plasma		Serum	
	Men	Women	Men	Women
N	30	29	32	25
Men/women	30/0	0/30	32/0	0/25
Age, y	59 ± 17	56 ± 16	56 ± 18	59 ± 14
TC, mg/dL	157 ± 28	175 ± 24	185 ± 39	189 ± 38
HDL-C, mg/dl	53 ± 11	67 ± 13*	53 ± 13	68 ± 15*
LDL-C, mg/dL	93 ± 21	95 ± 17	117 ± 36	103 ± 27
TG, mg/dL	87 ± 37	78 ± 23	102 ± 30	95 ± 39
sdLDL-C, mg/dL	17 ± 7	19 ± 8	27 ± 8	25 ± 12
Apo A-I, mg/dL	148 ± 19	170 ± 25	154 ± 28	182 ± 28
Antioxidative capacity of HDL (HII)	0.28 ± 0.04	0.30 ± 0.05	0.38 ± 0.06**	0.38 ± 0.05**

*p < 0.05, compared to men

**p < 0.05 compared to plasma

**Fig. 1:**
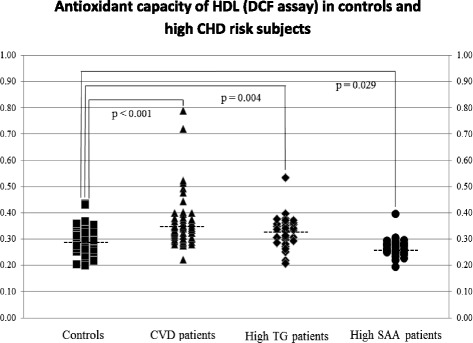
Distribution and median values of the antioxidant capacity of HDL as assessed by the DCF assay in apoBd plasma samples of apparently healthy subjects (Controls), patients with cardiovascular disease (CVD patients), patients with high triglyceride levels (High TG patients), and patients with high serum amyloid A levels (High SAA patients). Differences between groups were tested by Mann-Whitney *U* test

Characteristics of control subjects and patients whose DCF values were measured in apoBd plasma or serum are described in Table 2. All the lipid parameters in CHD patients and high TG subjects were significantly different from those in controls, except for apoA-I levels which were similar in controls and CHD patients. The CHD, high TG, and high SAA subjects had significantly higher TG levels than controls, and this was associated with lower HDL-C levels and altered distribution of HDL particles. Median DCF values in both CHD patients and high TG patients were significantly higher (indicating reduced antioxidative activity of HDL) than in controls (*p* < 0.001 and *p* = 0.004, respectively) (Fig. 1). Although high SAA subjects had lower HDL-C and higher TG levels than controls, their mean DCF value was significantly lower (better antioxidative activity of HDL) than that of controls (*p* = 0.03) (Fig. 1). In subjects with high MPO, serum lipids were similar to those of controls, with the exception of HDL-C (Table 2). The antioxidative capacity of HDL as measured in apoBd serum in high MPO subjects was similar to that of controls (0.356 ± 0.035 and 0.378 ± 0.056, respectively).

**Table 2:** Plasma or serum lipid profiles in control subjects and patients whose HDL antioxidative activities were measured in apoBd plasma or serum, respectively, with the DCF assay

	Plasma				Serum	
	Controls	CHD	High TG	High SAA	Controls	High MPO
Number	59	58	37	28	57	30
M/F, n	30/29	54/4**	25/12	19/9	32/25	15/15
TC, mg/dL	165 ± 26	145 ± 29**	262 ± 178**	163 ± 44	188 ± 38	169 ± 46
HDL-C, mg/dL	59 ± 14	43 ± 13**	34 ± 15**	51 ± 17*	60 ± 15	49 ± 16*
LDL-C, mg/dL	94 ± 19	80 ± 23**	115 ± 68*	89 ± 6	112 ± 35	100 ± 41
TG, mg/dL	74 (63–99)	128 (88–170)**	324 (264–1080)**	115 (92–163)*	97 (70–123)	116 (93–146)
sd LDL-C, mg/dL	16 (14–19)	37 (27–44)**	41 (27–63)**	17 (12–27)	25 (18–32)	23 (19–30)
Apo A-I, mg/dL	158 ± 23	152 ± 34	129 ± 45**	148 ± 48	168 ± 29	149 ± 33
Glucose, mg/dL	110 ± 17	103 ± 31	164 ± 80	110 ± 21	92 ± 15	105 ± 23
Insulin μIU/mL	13.2 ± 13.1	15.1 ± 9.5	19.8 ± 12.1	14.1 ± 7.2	8.3 ± 5.1	22.0 ± 13.5
Hs-CRP, mg/L	1.7 ± 1.9	2.4 ± 4.9	7.9 ± 15.1	5.5 ± 4.4	1.3 ± 1.2	2.7 ± 11.6
HDL distribution						
Preβ-1, %	8 (6–10)	12 (9–15)**	15 (10–19)**	8 (6–11)	5 (4–7)	5 (4–7)
α-1, %	23 (20–28)	15 (12–19)**	11 (7–17)**	17 (12–21)**	22 (19–25)	20 (16–22)
α-2, %	44 (42–47)	42 (39–45)*	41 (36–44)*	54 (49–61)**	41 (37–44)	40 (39–43)
α-3, %	15 (13–16)	18 (16–20)**	17 (13–23)**	11 (9–15)**	13 (12–15)	15 (13–17)
α-4, %	10 (8–11)	12 (10–14)**	13 (10–16)**	6 (5–9)**	11 (9–12)	12 (10–14)

Data are shown as mean ± SD or median (interquartile range). Differences in values between each group and Controls were tested by Pearson chi-squared test, Student-*t* test or Mann-Whitney’s *U* test (*p < 0.05 and **p < 0.01, relative to Controls)

### Correlations between HDL subpopulations and HDL antioxidative capacity

HDL’s antioxidative capacity was inversely associated with the relative (%) concentrations of the small preβ-1 and α-3 HDL particles (*r* = −0.162, *p* = 0.030, and *r* = −0.155, *p* = 0.037, respectively), and was positively associated with HDL-C concentrations and percent distribution of the intermediate size α-2 HDL particles (*r* = 0.198 *p* = 0.008 and *r* = 0.246, *p* = 0.001, respectively) (Table 2).

### Antioxidant capacity of HDL as measured by the DHR assay

**Fig. 2:**
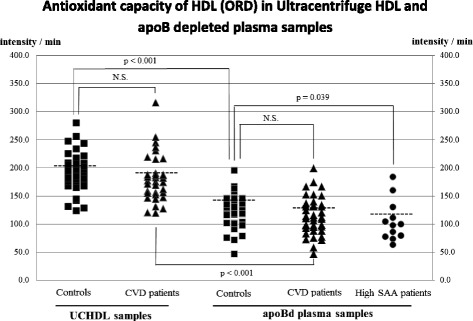
Distribution and median values of the oxidation rate of DHR (ORD) of ultracentrifuged HDL fractions (UCHDL) from apparently healthy subjects (Controls) and patients with the cardiovascular disease (CVD patients), and of apoB-deleted (apoBd) plasma from Controls, CVD patients, and patients with high serum amyloid A protein levels (High SAA patients). Differences between groups were tested by Mann-Whitney *U* test

**Fig. 3:**
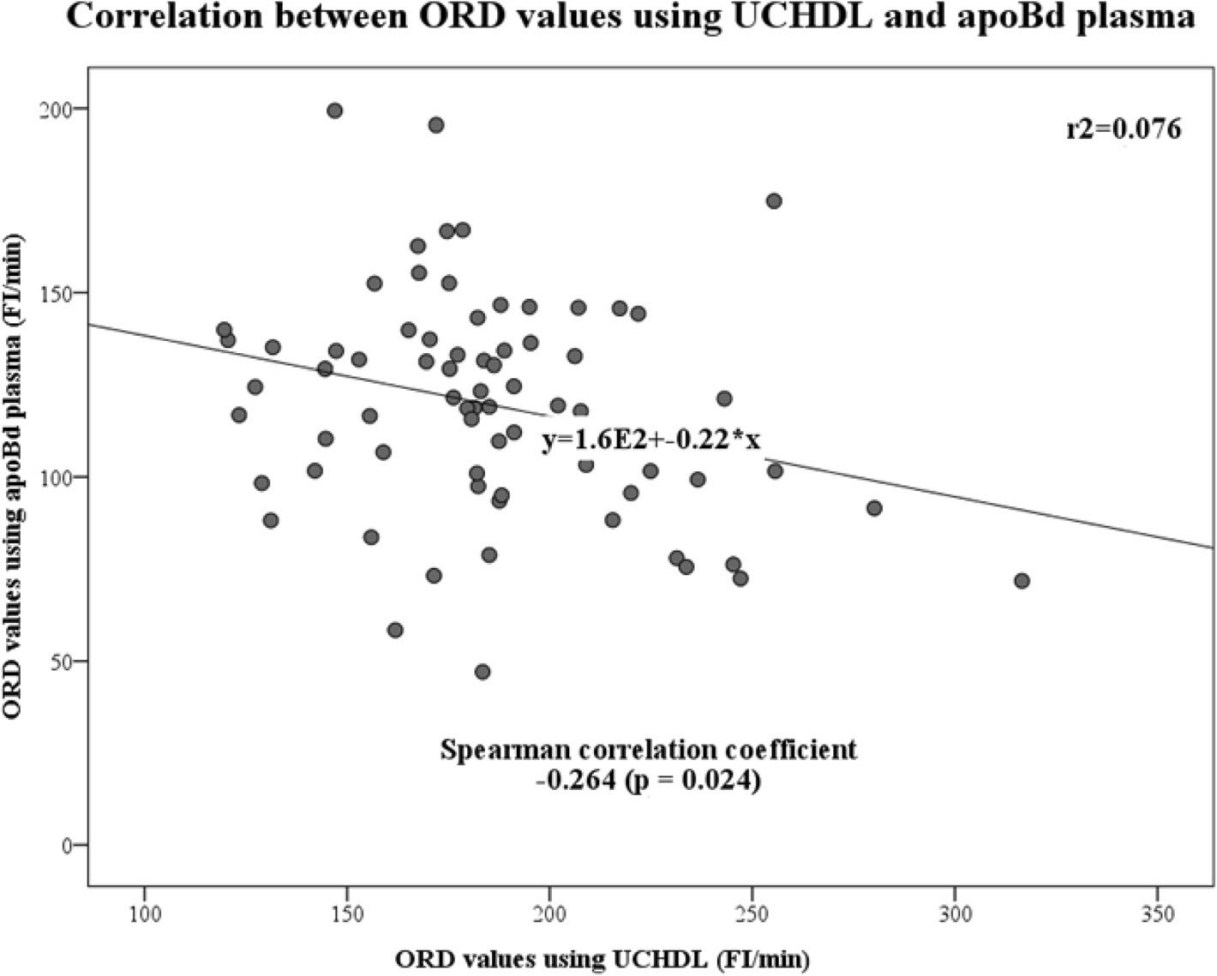
Correlation between the oxidation rate of DHR (ORD) of ultracentrifuged HDL fraction (UCHDL) and of apoB-depleted (apoBd) plasma in apparently healthy subjects and patients with cardiovascular disease

Plasma lipid profiles of the subjects whose oxidation rate (ORD) were measured with the DHR assay are shown in Table 3. HDL-C and apoA-I levels were lower in CHD patients than in controls. Plasma lipid profiles of high-SAA subjects were not significantly different from controls. ORD was measured using both UCHDL and apoBd plasma in control and CHD subjects. The median level of HDL antioxidative capacity was similar in controls and CHD patients, whether measured using UCHDL or apoBd plasma samples (Fig. 2). However, the antioxidant capacity was significantly higher in UCHDL than in apoBd plasma (both *p* < 0.001) (Fig. 2). High SAA subjects had significantly lower median ORD value (better antioxidative activity of HDL) compared to controls. Unexpectedly, there was an inverse association between the ORD values obtained from UCHDL and apoBd plasma (Spearman correlation coefficient: −0.264, *p* = 0.024) (Fig. 3).

**Table 3:** Plasma lipid profiles of control subjects and patients whose HDL antioxidative capacity was measured in ultracentrifuged HDL (UCHDL) and apoBd plasma samples using the DHR assay

	Controls	CHD	High SAA
Number	41	40	12
M/F, n	14/27	22/18	7/5
TC, mg/dL	174 ± 36	171 ± 41	193 ± 79
HDL-C, mg/dL	66 ± 18	53 ± 17**	72 ± 58
LDL-C, mg/dL	102 ± 27	99 ± 36	99 ± 44
TG, mg/dL	93 (68–122)	103 (77–153)	84 (62–133)
sd LDL-C, mg/dL	23 (17–29)	38 (23–45)	27 (17–32)
Apo A-I, mg/dL	172 ± 29	156 ± 33*	168 ± 78

Data shown as mean ± SD or median (interquartile range). P value of the male-to-female ratio between the patients with cardiovascular disease and the control subjects is 0.059 analyzed by chi-squared test

*p < 0.05, **p < 0.01 compared to controls

## Discussion

It has been proposed that HDL can protect against atherosclerosis by reducing LDL oxidation and protecting cells from oxidative stress, important players in the development and progression of atherosclerotic lesions [14]. A number of studies using the DCF assay have demonstrated that patients with chronic inflammatory diseases have lower antioxidative capacity (higher DCF values, expressed as HII) compared to healthy subjects [15–17]. Recently, it has also been reported that higher HII values are associated with increased mortality in critically ill patients [18]. On the other hand, another study has shown that, while acute coronary syndrome patients had significantly higher than normal HII values, no differences were observed between chronic CHD patients and healthy subjects [5]. These results suggest that the DCF assay can detect alterations in the antioxidative potential of HDL in some, but not all patient populations.

Similarly, in our study, we have shown that the antioxidative capacity of HDL assessed by the DCF assay is significantly lower in CHD patients (higher HII) than in healthy subjects. High TG levels were also associated with decreased antioxidant capacity. However, it should be noted that none of the CHD subjects in our study had a DCF value, or HII index, above 1, a set limit of pro-inflammatory HDL. Moreover, other conditions that are believed to be associated with dysfunctional HDL, such as elevated SAA or MPO levels were not associated with impaired antioxidative capacity [19]. Actually, subjects with elevated SAA levels showed a higher antioxidant capacity than controls. Similarly to other studies, our study documented a large overlap in the measures of antioxidative activities of HDL among controls and diseased subjects.

HDL particles are heterogeneous in size, composition and function [12]. A number of studies have revealed that various HDL subpopulations are differently associated with the antioxidative activity of HDL. Kontush et al. [20, 21] demonstrated that in normolipidemic subjects, the smaller HDL3 fraction had more favorable biological and functional properties than the larger HDL2 fraction and that the antioxidative activity of HDL was predominantly associated with HDL3. Other researchers have shown that, in diabetic patients, unfavorable changes in the chemical composition and enzyme activities of HDL3 are associated with reduced antioxidative activity of HDL [22, 23]. Our data indicating a significant correlation between and HDL-C and HDL antioxidant capacity is in line with others’ data [5]. When we measured HDL subpopulations, we observed an inverse correlation between DCF and α-2 HDL particles. This HDL subpopulation is found mostly in the HDL3 fraction [23]. Therefore, our findings are in agreement with previous studies and support a specific involvement of the normally most abundant HDL particle, α-2 in the antioxidative capacity of HDL. Interestingly, LDL-C levels also correlated inversely with DCF value: it is possible that statin use in the CHD patients explains this unexpected result, since statin therapy has been previously shown to improve DCF values [5]. In contrast to total LDL-C, sdLDL-C, the most atherogenic fraction of LDL, had a positive correlation with DCF values.

Our data also indicate that DCF values were higher in serum than in plasma samples. This difference may be explained, at least in part, by the antioxidant effects of fibrinogen [24]. This raises the question whether we can measure HDL’s antioxidative capacity in apoBd samples which contains a large number of other potentially redox compounds. On the other hand, it is well documented that HDL goes through significant alteration during ultracentrifugal separation, which might alter its redox capacity.

When the DHR assay was used to measure HDL antioxidative capacity, we did not observe a significant difference between the median values in controls and CHD patients. A previous study using the DHR assay has shown that, although HDL from patients with psoriasis had lower cholesterol efflux capacity than control subjects, there was no difference in the HDL antioxidant capacity between the two groups [25]. Kelesidis et al. [26], who had developed the DHR assay, have described some of the limitations of the assay and reported that the ORD value is susceptible to not only environmental factors but also to factors associated with lipoproteins, such as type and concentration of each lipoprotein particle, profile of apolipoproteins, and lipoprotein-isolation methods. For example, the weight of added LDL, a pro-inflammatory lipoprotein particle, correlated significantly and inversely with the DHR value. Moreover, in our study, the median ORD value from UCHDL samples was significantly higher than that of apoBd samples: furthermore, the ORD values of UCHDL samples correlated negatively with those of apoBd samples. Similarly, Kelesidis et al. have observed differences between UCHDL and apoBd serum values, as indicated by median ORD value in patients infected by HIV being significantly higher than in healthy subjects when using apoBd serum samples but lower when using UCHDL samples [26]. Based on these data and the results of our study, DHR should be considered an unsuitable fluorescent probe for the measurement of the antioxidative activity of HDL.

SAA, an acute-phase protein primarily synthesized by hepatocytes, replaces apoA-I in HDL and forms monodisperse (α-2) HDL particles. A number of experimental studies have suggested that SAA can accelerate atherosclerosis possibly by impairing the antioxidative capacity of HDL [27, 28]. In contrast, our study indicated that patients with high SAA levels had significantly lower ORD or DCF values (better antioxidative activity of HDL) than controls.

A limitation of our study was that we did not have complete information on medical histories and medications in all of our study subjects. Another potential limitation is that the number of the subjects might be too small to yield significant results about the DHR assay.

## Conclusions

We are led to the following conclusions: 1) the antioxidant capacity of HDL is affected by the medium: apoB-depleted plasma and serum, and ultracentrifugally isolated HDL exert different antioxidant capacity; 2) although the DCF assay could detect significant differences in the antioxidative activity of HDL between controls and patients, the practical use of this assay is limited by the large overlap in values among groups; 3) the DHR assay failed to measure accurately the antioxidative activity of HDL, possibly due to the susceptibility of the probe to various factors; and 4) the antioxidative activity of HDL in patients who have elevated SAA levels needs to be reassessed.
